# Photonic Nano-/Microstructured Diatom Based Biosilica in Metal Modification and Removal—A Review

**DOI:** 10.3390/ma15196597

**Published:** 2022-09-23

**Authors:** Piya Roychoudhury, Rahul Bose, Przemysław Dąbek, Andrzej Witkowski

**Affiliations:** 1 Institute of Marine and Environmental Sciences, University of Szczecin, Mickiewicza 16a, 70-383 Szczecin, Poland; 2 Department of Botany, University of Calcutta, Ballygunge Circular Road 35, Kolkata 700019, India

**Keywords:** diatom, frustules, silica-metal conjugate, absorption, adsorption

## Abstract

The siliceous exoskeletal shells of diatoms, commonly known as frustules, have drawn attention because of their photoluminescence property and high volume to surface area. Photonic biosilica can also enhance the plasmonic sensitivity of nanoparticles. Because of this, researchers have studied the effectiveness of various metal particles after combining with biosilica. Additionally, naturally occurring diatom-based biosilica has excellent adsorption and absorption capabilities, which have already been exploited for wastewater treatment. Moreover, the nanoporous, ultra-hydrophilic frustules can easily accumulate more molecules on their surfaces. As a consequence, it becomes easier to conjugate noble metals with silica, making them more stable and effective. The main focus of this review is to agglomerate the utility of biocompatible diatom frustules, which is a no-cost natural resource of biosilica, in metal modification and removal.

## 1. Introduction

Diatoms are considered a major group of phytoplankton that accounts for approximately 40% of ocean carbon fixation and is responsible for most biosilica production through the construction of their cell walls. These microorganisms are used in the synthesis of pigments, enzymes, biofuels, biosilica, mineral materials, etc. [1,2,3]. Interestingly, almost all diatom cells are encased by a siliceous skeleton called frustule. Light microscopy was the primary tool for the study of diatoms; however, with the discovery of intricacies, symmetry, and apparent stability of their wall patterns, frustule morphology has become the basis of their identification and classification. Based on the frustules structure, diatoms are of two types—centric and pennate. Pennate diatoms are found in equal number in both fresh and marine habitats, whereas centric diatoms dominate the marine environment. When these organisms die, the constant rain of dead diatom frustules over the highly productive parts, i.e., the bottom of ocean, results in the accumulation of siliceous oozes, which over the time become fossilized and are termed as diatomaceous earth (DE) [4].

Biogenic or opaline silica, also known as hydrated silica (SiO_2._nH_2_O), makes up the diatom frustule [5]. It accounts for 97% of all dissolved silicon. The frustule consists of two halves: hypotheca and epitheca, that are kept together by girdle bands, and which are likewise patterned silica structures. These girdles wrap around the diatom’s whole circumference, and together with hypotheca and epitheca, make up the entire cell wall. Many microscopic pores and perforations in the cell walls allow the membrane to contact and exchange small molecules with the outside world. Between generations, these complex and nanoporous cell walls are nearly identical. Every species has its own set of frustules.

Biosilicification is the process by which diatoms create their elaborate and decorative siliceous frustules. Although there are several competing theories, research continues to improve our understanding of this often-perplexing process. Some parts of the process remain obscure. Diatoms are so good at absorbing silicon from their surroundings that they can almost deplete silicon levels to the point where ordinary water monitoring techniques cannot detect it [6]. They require silica from their surroundings in order to create frustules. In moist or watery conditions, soluble silicon is present in the form of orthosilicic acid (Si(OH)_4_) [7]. Silicon enters the diatom from the aqueous environment in the form of silicic acid [8] via specific transmembrane proteins (SITs: Silicic acid transporters) [9,10]. Silica polycondensation and assembly occurs in specialized vesicles known as silica deposition vesicles (SDV) mainly by sillafins and Long Chain Polyamines (LCPAs) together with the cytoskeleton, which is closely bound to the cytosolic surface of the silicalema [11,12], where silica can be accumulated at concentrations up to 1000× higher than in the surrounding aqueous environment [13]. Silaffins and LCPA accelerate and control silica morphogenesis from silicic acid and are believed to be involved in morphogenesis of the species-specific nanopatterns in the SDV.

Diatom populations are frequently silica-limited [14] and will “bloom” if their surroundings are enhanced by silica-rich run-off. If silica is reduced, their population will plateau before dropping, allowing other algae that are not silicate-dependent to take over. Diatoms use silica as structural material and fabricate silica shells in a bottom-up self-assembly process generating nanosilica as building blocks [15]. Due to these features and qualities, diatoms are considered unique machines for nanosilica biosynthesis. The recent development of synthetic nanotechnology has been driven by the nearly insatiable global demand for ever-smaller structures for electronic, optical, chemical, or biomedical devices [16]. Therefore, the diatom mediated eco-friendly, cost-effective process has become popular among nanotechnologists for the production of an exceptional variety of patterned silica nanostructures. These hierarchical biosilica can also act as a photonic nano-platform. The porous siliceous skeleton emits colorful transmission spectra after irradiation by different wavelengths. When frustules of *Coscinodiscus* sp. have been irradiated by UV-wavelengths, these have emitted one or more photoluminescence peaks in the visible regions [17,18]. The photonic properties have also been observed in pennate frustules of *Melosira varians* [19]. Additionally, frustules show a light filtering ability by which they can protect diatom cells from harmful light [17,19]. During capturing of the fluorescence image of *Thalassiosira rotula* under irradiation (325 nm) of a high-pressure mercury lamp, frustules of the diatom being photoluminescent showed multiband emissions between 450–690 nm. It has also been reported that the surrounding environment affects the photoluminescence property of *Thalassiosira rotula* [20]. The effect of nitrogen dioxide (NO_2_) on the photoluminescence property of marine diatoms viz. *Coscinodiscus wailesii* and *Thalassiosira rotula* have been studied, which confirmed that the photoluminescence signal was quenched with electrophilic NO_2_ as electrophilic gases can attract some electrons from the silica skeleton [17,21]. The photoluminescence properties of centric and pennate diatoms like *Coscinodiscus wailesii, Actinoptychus senarius*, and *Cocconeis scutellum* have already been utilized for gas detection [22]. The photonic property was also reported in chemically synthesized silica-based particles [23,24].

Due to their unique optical properties and large surface area, frustules have been used by many researchers for fabrication of various silica-material nano-/micro-composites. The valve surface of diatoms can easily be modified by linking with other materials due to the presence of reactive silanol (Si–OH) groups all over the surface [25]. Exploiting this property, the siliceous ornaments of diatoms have already been loaded with nucleic acids to fabricate a potential substrate for surface enhanced Raman spectroscopy (SERS) [26]. The frustules of *Aulacoseira* sp. have been functionalized with dopamine iron oxide [27], organosilanes and phosphonic acid [28], graphene oxide [29], phospholipid [30], and oligo (ethylene glycol) methacrylate [31] to deliver the drugs named indomethacin, gentamicin, indomethacin, carbamazepine and levofloxacin, respectively. The calcined diatomite has also been modified with 3-aminopropyl-triethoxysilane and N-(γ-Maleimidobutyryloxy) sulfosuccinimide ester (ATPES-sulfo-GMBS) for delivery of SiRNA [30]. The chemically changed surfaces of *Thalassiosira weissfloggi* and *Nitzschia* sp. showed efficacy in loading and releasing of anti-cancer drugs like ciprofloxacin [32] and curcumin [33], respectively. Moreover, these siliceous structures could be used as templates in the patterning of molecules at the nano-microscale because of their intricate structural geometries. In this review, alteration of diatom-based biosilica with various metals like titanium, germanium, palladium, platinum, silver, gold, calcium, manganese, iron, cadmium, zinc, aluminium, nickel, europium, zirconium and tin to generate more progressive functional materials have been described with their potential applications.

Diatoms have been employed to detect a variety of toxic pollutants in rivers, lakes, and other water bodies because of their diverse habitat range and survival potency with excessive growth rate in extreme environmental conditions. The utmost adsorption (biosorption) and absorption capabilities of their siliceous shells play a major role in accumulation of harmful elements. For these reasons, diatomaceous earth has been utilized in various environmental and commercial applications including water purification, dye adsorption, heavy metal removal, noble metal extraction and waste degradation processes [34,35,36,37,38,39,40,41,42,43]. Additionally, diatoms are highly tolerant to metals. Their high metal uptake capacity is well recognized and validated [44,45,46]. The perforated, hydrophilic frustules can accommodate a high amount of toxic elements within a single cell. For gold bio-recovery tests, Chakraborty et al., 2006 [47], used two diatom strains, *Navicula* and *Nitzschia*. The interactions between various metal ions viz. arsenic, chromium, mercury, silver, lead, copper, calcium, zinc and external covering of diatoms have been summarized in this review, emphasizing the harmful metal removal abilities of diatom-based biosilica.

## 2. Fabrication of Metal-Silica Nanocomplex Using Diatom Based Biosilica

**Figure 1:**
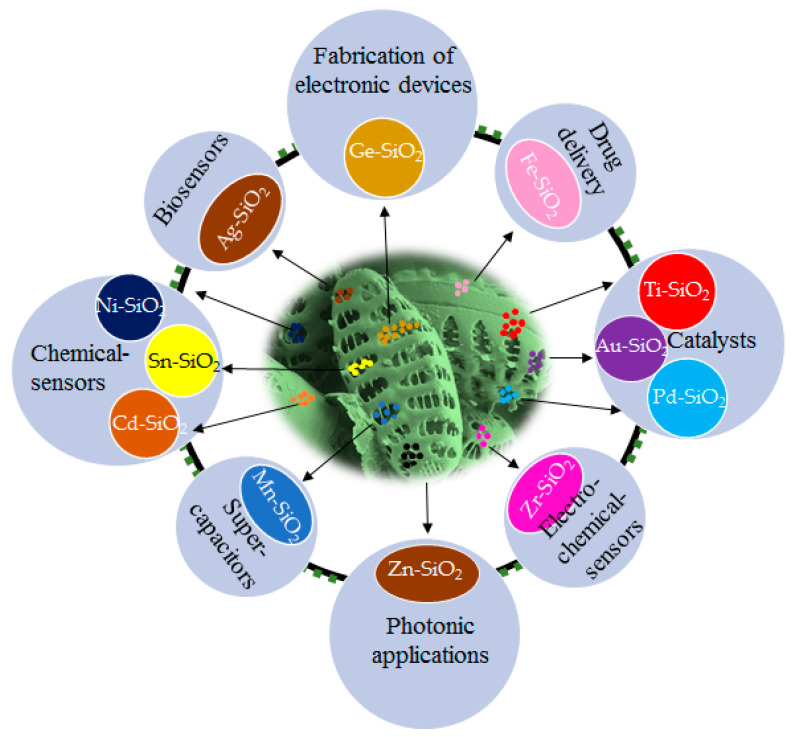
Applications of metal conjugated siliceous frustules.

Researchers have described some methods like sol-gel, laser ablation, etc., to produce silica particles in laboratory condition [48,49,50]. One of the main advantages of diatoms exploitation in nanobiotechnology is the cost-effectiveness of obtaining complicated structures like three-dimensional (3D) nano-/microstructures that self-replicate at high rates with outstanding reproducibility, resulting in complex geometries and architectures that are difficult to achieve even with the most sophisticated fabrication techniques [51,52,53]. However, silica may not be the best choice for certain applications. Various approaches have been devised over the years aiming at the fabrication of realistic frustule replicas following an eco-friendly route by metabolic incorporation of metal ions in diatom cells, which does not allow the total replacement of silica. However, silica associated metal particles are more acceptable in catalysis. Nowadays, immobilization of noble metals like gold, silver, etc., to construct active and stable catalysts on solid support is the focus of significant research [54,55]. DE is commonly employed as an ideal support in the construction of hierarchical porous catalysts due to its porous structure, low density, large surface area, and abundant sources [56,57,58,59]. All possible applications of metal-doped diatom frustules are shown in Figure 1 and Table 1.

### 2.1. Titanium, Germanium, Palladium and Platinum

*Pinnularia* sp., a rod-shaped diatom, was used as a platform for the biological manufacturing of a silica–titania (Si-TiO_2_) composite material in which nanosized TiO_2_ was inserted physiologically [59]. Diatom frustules–TiO_2_ composites have been extensively studied and utilized in recent years to improve dye-sensitized solar cells (DSSCs) efficiency [89,90,91,92,93]. In DSSCs, TiO_2_ was replaced by TiO_2_ particle loaded frustules after plasma treatment [59]. The resulting 3D hybrid structures have a large effective surface area, and the multiple scattering events caused by the frustule’s pores ensured that the incoming light interacts with dye electrons more effectively. In comparison to traditional DSSCs, a 30 percent boost in conversion efficiency was achieved after only three cycles of plasma treatment. According to some studies, soluble titanium (Ti) in the form of titanium(iv)-bis ammonium-lactato-dihydroxide (Ti-BALDH) was directly taken up from the aqueous solution and incorporated into the frustule (girdle band) by *Thalassiosira weissflogii* at a ratio of 0.34:1 (Si:Ti). Since diatoms have potential enough to grow and divide in Ti-spiked growth medium, high-solubility Ti substrates can thus be used for biomineralization in diatom cells [66]. Basharina et al., 2012 [67] performed a one-stage doping process to integrate Ti into the frustules of *Synedra acus* in a microincubator where 10 mM sodium silicate (Na_2_SiO_3_) and 10 mM titanium tetrachloride (TiCl_4_) were mixed into the growth medium. Many researchers have used a two-way doping process to incorporate Ti into the frustule of various diatoms like *Pinnularia* sp. [94], *Coscinodiscus* sp. [68], and *Fistulifera solaris* [69].

In another experiment, *Pinnularia* frustules with biologically incorporated germanium oxide (GeO_2_) acted as a luminous source, which was used to develop an electroluminescent device [70]. Blue photoluminescence (PL) is known to exist in native biosilica [72,95,96,97], and the diatom–GeO_2_ devices showed both PL and electroluminescence (EL) properties. The frustules consisting of metabolically implanted GeO_2_ exhibited two bands of emission when a voltage was given to the device, the first between 300 and 500 nm, and the latter between 640 and 780 nm. The introduction of GeO_2_ into the biosilica of *Pinnularia* sp. changed the pore structure of the frustule [71]. The expected photonic features of the frustule pore lattice were compatible with the emission bands from the device when the GeO_2_-modified frustules were represented as photonic crystals [98,99]. Diatom frustules with tunable elements can thus be utilized to develop any desired optoelectronic features. Qin et al., 2008 [72], also developed a photoluminescent Ge-Si nanocomb utilizing *Nitzschia frustulum* as source of biosilica. The final output of the two-stage culture procedure of *Nitzschia frustulum* comprised 0.41% wt Ge in biosilica and was made up of an equal mix of parent frustule valves with a regular two-dimensional arrangement of 200 nm pores and daughter valves with the nanocomb structure. Simultaneously, Davis and Hildebrand, 2008 [73], proved that Ge at lower levels did not alter the valve morphology, whereas at higher levels, it did modify the frustule structure of *Thalassiosira pseudonana*. These initiatives are the initial steps towards incorporating Ge into diatom cells and consequent development of photoluminescent advanced nano-/micro structures for electronic devices. However, further research is needed for the development of green micro- and nanoelectronic components.

**Figure 2:**
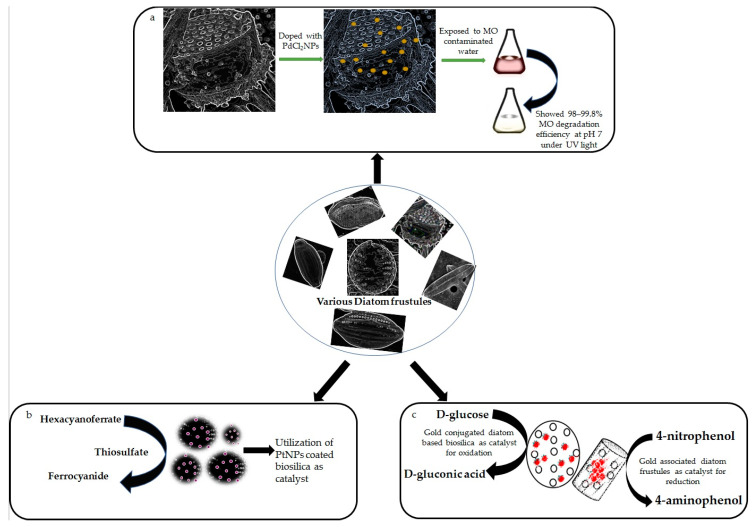
Showing catalytic activities of various metal associated diatom-based biosilica. (**a**) Schematic diagram on methyl orange removal by palladium chloride doped biosilica of *Pseudostaurosira trainorii*, observed by Sprynskyy et al., 2021 [75]. (**b**) Platinum nanoparticles coated frustules showed 10 times more catalytic efficiency in redox reaction between hexacyanoferrate(III) and thiosulfate than platinum collloids, confirmed by Jantschke et al., 2012 [76]. (**c**) Catalytic abilities of AuNPs loaded biosilica in oxidation and reduction have been documented by Fischer et al., 2016 [63] and Yu et al., 2010 [64] respectively.

Due to the distinctive hierarchical pore structure, diatom biosilica is an appealing support of nanoparticles (NPs) for catalytic purposes [100]. The diatomite–palladium (Si-Pd) composites exhibited catalytic efficacy in Heck and Suzuki reactions [74]. Similarly, in another study, a new catalyst based on biosilica of *Pseudostaurosira trainorii*, doped with palladium (II) chloride particles (PdCl_2_NPs), was prepared and tested for efficient degradation of methyl orange (MO) in a water solution under UV light excitation [75]. A schematic diagram on MO degradation by PdCl_2_NPs-doped biosilica has been shown in Figure 2a.

Since diatomite is a readily available, low-cost industrial commodity with high catalytic activity and, therefore, the catalyst may be separated with ease, and such composite materials are intriguing prospects for industrial applications. That is why the catalytic capabilities of platinum (Pt) NPs coated *C. wailesii* cell walls were also investigated against redox reaction between hexacyanoferrate (III) and thiosulfate [76] (Figure 2b). The absorbance at 420 nm was used to determine the concentration of hexacyanoferrate-(III). The reaction rate in the presence of Pt-coated biosilica was 15–25 times higher than that of pure biosilica and 10 times more than Pt colloid, demonstrating that PtNPs remain catalytically active even when linked to diatom cell walls. It was also mentioned by the authors that PtNPs coupled diatom biosilica even had higher specific catalytic activity (approximately 2 times) than Pt–fungus hybrids [76].

### 2.2. Silver

**Figure 3:**
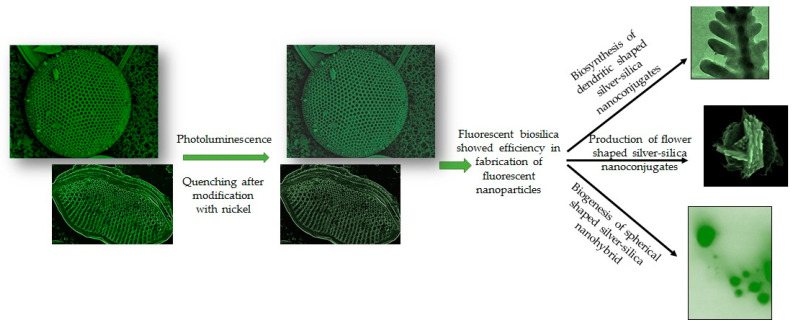
Schematic representation on biogenesis of biosilica-inspired fluorescent nanostructures. Representation of reduced photoluminescence in diatom frustules after modification with nickel as reported by Townley et al., 2007 [85]. Depiction of diatom mediated Production of dendritic; flower and spherical shaped, fluorescent silver-silica nanoconjugates as reported by Bose et al., 2021 [60] and Roychoudhury et al., 2021 [102] respectively.

Silicious frustules of *T. weissflogii* have been decorated with silver nanoparticles (AgNPs) exploiting the adhesion properties of polydopamine (PDA) [101]. Similarly, a dendritic shaped Ag-SiO_2_ 3D nanostructure has been developed biogenically by Bose et al., 2021 [60]. The authors described an eco-friendly, simple technique to biosynthesize dendritic shaped Ag-SiO_2_ nanohybrid utilizing nanoporous frustules of *Halamphora subturgida* as reducing agents. This method confirmed that biosilica is an effective reducing agent due to the presence of Si-OH groups and capable in synthesis of crystalline Ag-SiO_2_ nanohybrid after exposure to 9 mM of silver nitrate solution for 72 h without involving any hazardous chemical [60]. Synthesized dendritic nanostructures showed fluorescent property. Similarly, the diatoms *Gedaniella flavovirens* and *G. mutabilis* have been identified as efficient reducing agents for the production of flower and spherical shaped fluorescent Ag-SiO_2_ nanohybrids [102]. Diatom inspired various shaped fluorescent Ag-SiO_2_NPs have been shown in Figure 3.

**Figure 4:**
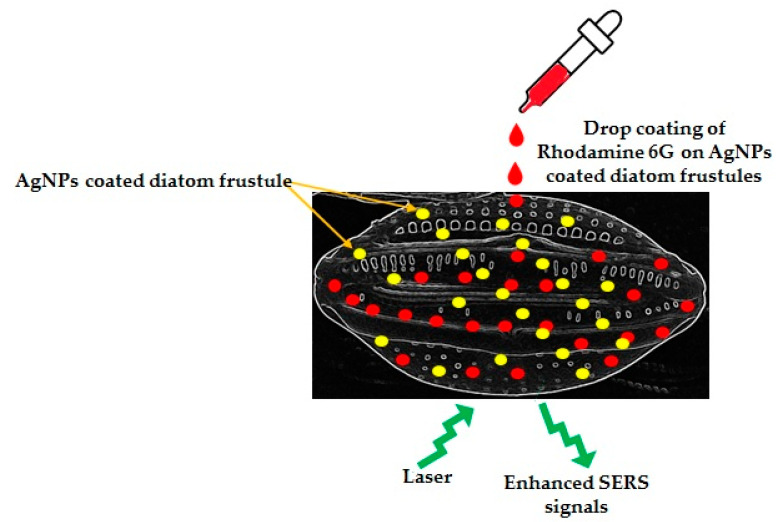
Schematic representation of enhanced SERS signal with 2 times stronger optical extinction and 4 times higher sensitivity of Rhodamine 6G while using AgNPs loaded diatom frustules as a substrate, recorded by Ren et al., 2013 [61].

It was also observed by many researchers that diatom frustules acted as an integration platform to enhance localized surface plasmon resonances of self-assembled AgNPs on the surface of diatom frustules. Ren et al., 2013 [61], fixed AgNPs on the frustules of *Pinnularia* sp. using aminopropyltriethoxyl-silane (APTES) as an adhesive. The AgNPs-on-biosilica showed 2 times stronger optical extinction and 4 times higher sensitivity in surface-enhanced Raman scattering of Rhodmine 6G than the AgNPs-on-glass structure (Figure 4). The porous frustules of *Pinnularia* sp. have also been exploited by Sivashanmugan et al., 2019 [103], to prepare photonic crystal-enhanced plasmonic mesocapsules of biosilica and AgNPs to achieve ultrasensitive sensing in optofluidic-SERS. It is well known that the porous structures of diatom frustules are able to carry a high amount of NPs, and the submicron pores are very effective for capturing analytes. Therefore, Sivashanmugan et al., 2019 [103], decorated diatom biosilica with in situ grown AgNPs for high density AgNPs assemblage on the porous diatom biosilica frustules. These unique mesocapsules showed 100 times more enhancement factors and a 1000 times higher detection limit compared to colloidal AgNPs in optofluidic-SERS. Korkmaz et al., 2018 [104], designed simple, inexpensive SERS strips with diatom frustules and AgNPs. The SERS strips showed a nine times higher enhancement factor than the AgNPs-on-glass strips. This strip also showed great potential to detect and identify bacteria. Kraai et al., 2020 [105] developed ultrathin layer chromatography (UTLC)-SERS plates combining AgNPs on the porous surface of *Pinnularia* sp. frustules for detection of different analytes.

### 2.3. Gold

Polyethylene glycol (PEG) modified porous diatomite has been decorated with gold nanoparticles (AuNPs) [62] following one-plot-liquid phase method. The designed Au conjugated diatomite with an average size of 450 nm could be considered as a safe material for medical applications like imaging and drug delivery as the nanocomplexes (100 μg ml^−1^) showed 90% cell viability of HeLa cells after 72 h of incubation. In another experiment, biosilica from three different diatoms (*Stephanopyxis turris*, *Eucampia zodiacus*, and *Thalassiosira pseudonana*) were loaded with AuNPs using a covalent coupling method [63]. The biosilica showed very high Au-loading capacities (up to 45% wt), with a homogeneous NPs distribution. The Au loaded surface provided a highly catalytically active surface and has been exploited as a favorable catalyst for the oxidation of d-Glucose to d-gluconic acid.

In another study, 3D Au-nanostructures were created by employing electroless deposition of Au onto diatom-derived silica substrates. These materials showed a significant catalytic property in reduction of 4-nitrophenol to 4-aminophenol in the presence of reductants, such as sodium borohydride (NaBH_4_) [64]. The catalytic abilities of AuNPs loaded biosilica in oxidation and reduction have been shown in Figure 2c. The frustules of *Pseudostaurosira trainorii* have been decorated with AuNPs for the detection of interleukin 8 (IL-8) in blood plasma by an ultrasensitive SERS immunoassay [106]. Interestingly, Kong et al., 2017 [107], devised a facile lab-on-chip device with ultra-sensing capabilities by combining plasmonic diatomite with AuNPs for on-chip chromatography and label-free SERS sensing. Recently, researchers [108] created a nanoplatform with diatomite and AuNPs to release an anticancer drug against colorectal cancer. The nanohybrid has been encapsulated with the anticancer drug galunisertib, and the release of the drug was monitored by SERS. Similarly, the chemical modification of biosilica derived from *Aulacoseria* sp. with AuNPs has been performed [65] to understand their capabilities in loading and releasing gentamicin in simulated body fluid.

Layer-by-layer deposition or covalent attachment of AuNPs to the biosilica surface were both successful methods for creating diatom-templated Au arrays [76]. The AuNPs loading on frustules of *Thalassiosira pseudonana* could be improved by previous functionalization with APTES [109]. Alternatively, Au sputtering can be used, however sputtering can cover structural features of samples, and even the presence of AuNPs with a diameter of only 4 nm can effectively inhibit the charge-induced picture distortions as seen in case of the carbon-sputtered sample. However, in the AuNPs coated sample, minute structural elements of the cell wall are considerably more evident. Therefore, to eliminate charge-induced picture distortions in scanning electron microscopy (SEM), AuNP coating might be recommended as a simple and inexpensive alternative to routinely utilized sputtering procedures [110].

### 2.4. Calcium

Leone et al., 2017 [83], recently presented findings on calcium ions (Ca^2+^) spiked diatomaceous biosilica for biomedical materials. The research was inspired by the fact that fibroblasts and osteoblasts grow effectively on silica or ceramic substrates and the presence of Ca^2+^ encourages cell growth. *Thalassiosira weissflogii* was cultured in autoclaved and ultrafiltered sea water at a regulated temperature of 18–22 °C with the inclusion of Ca^2+^ in the form of calcium chloride (CaCl_2_, 14 mM) to produce Ca-doped diatomaceous biosilica. The introduction of Ca^2+^ to diatom culture medium showed little effect on their form or structure, according to SEM. Fourier-transform infrared spectroscopy (FTIR) revealed that, despite the activity of 30% hydrogen peroxide to eliminate organic materials from diatom cells, Ca remained in the frustules. Ca concentration (0.9 ± 0.05% wt) in the modified frustules was determined by Energy Dispersive X-ray analysis (EDAX). The authors concluded that Ca-doped biosilica can be used as an effective substrate for the growth of fibroblasts and osteoblasts, which could be useful in regenerative medicine.

Li et al., 2018 [84], also investigated Ca^2+^ doping on frustules of *Coscinodiscus* sp. At 21 °C, *Coscinodiscus* sp. was cultivated using an ultrafiltered and autoclaved f/2 Guillard medium, and Ca incorporation in diatom frustules was performed by adding CaCl_2_ to the culture medium. X-ray Diffraction (XRD) and EDAX studies confirmed the presence of Ca^2+^ in the structure of diatom frustules. The presence of Ca^2+^ in the growth medium did not produce any major changes in the morphology of diatoms according to Li et al., 2018 [84], and the authors suggested that the obtained material may be utilized as a haemostatic, similar to Leone et al., 2017 [83].

### 2.5. Manganese, Iron, Cadmium, Zinc

Li et al., 2019 [77], developed manganese–iron oxide (Mn-FeOx) hybrids with diatomite. The produced hybrid replicas retained the structure and morphology of the original frustule. A wide range of materials (MnFeOx, Fe (OH)x, and FeO-OH) and their copies were examined as electrodes in asymmetric supercapacitors where MnO_2_ replicas behaved as positive electrodes and FeO-OH replicas behaved as negative electrodes. The increased surface area and active patches in contact with the electrolyte improved the electrochemical properties of the investigated electrodes. The intrinsic features like permeability, high surface area, and cytocompatibility of functionalized diatoms’ biosilica make it particularly appealing for drug delivery applications. Magnetic iron oxide nanoparticles (Fe_3_O_4_) modified with dopamine were covalently fixed on the surface of diatomaceous earth, resulting in magnetically directed microcarriers for indomethacin drug delivery [27].

Semiconducting cadmium sulphide (CdS) NPs were produced on the surface of *Pinnularia* sp. frustules using a chemical-bath deposition (CBD) approach owing to the batch reaction of cadmium chloride (CdCl_2_) and thiourea as cadmium and sulphide ions, respectively [78]. The generated frustules were compact, homogenous, and nanostructured, with a bright yellow emission in their photoluminescence spectra, which is characteristic of CdS nanoparticles. These findings have paved the way for usage of new nanostructured materials with controllable photoluminescence in photodetectors, solar cells, and a variety of other optoelectronic devices.

Cai et al., 2007 [80], observed that immersing biosilica shells of diatomaceous earth in a solution of zinc acetate dihydrate and manganese acetate resulted in a manganese–zinc orthosilicate (Mn-Zn_2_SiO_4_) coating on diatom frustules. Even after filtering and heating at 1050 °C, the frustules were covered with a compact, uniform and green-emitting Mn-doped Zn_2_SiO_4_ layer, suitable for photonic applications. In another experiment, the biosilica of *Coscinodiscus lineatus* after coating the exterior surface with high-refractive-indexed zinc sulphide (ZnS) NPs was utilized for optical as well as photonics applications because of the increased dielectric contrast between ZnSNPs and air phases. The process was done using an ultrasound-based approach [79], keeping their shape and photonic-crystal features intact.

### 2.6. Aluminium, Nickel, Europium, Zirconium and Tin

Machill et al., 2013 [81], investigated the incorporation of aluminum (Al) into the frustule of *Stephanopyxis turris* using artificial seawater with various concentrations of aluminum chloride (AlCl_3_) viz. 10.5, 42.5, 105.5, and 1055 µm. At these concentrations, the mass ratios of Al:Si were 1:10, 1:2.5, 1:1, and 10:1, respectively. To avoid uncontrolled Al precipitation, Al was added into the medium in the form of bis–tris-chelates. Comparing control diatoms that were grown in the Al free culture medium to the treated ones, no significant morphological changes were observed by SEM analysis. Frustules in both Al-enriched and control diatom samples were of same size and shape. The concentration of Al was determined in biosilica by inductively coupled plasma-optical emission spectrometry (ICP-OES). Quantification has shown that the amount of Al embedded in frustules increased significantly in Al-enriched media. The ratio of Al:Si in cell walls was discovered as 1:15. Diatomaceous biosilica doped with Al ions can thereby become a desired material for catalysis due to its strong catalytic activity [82].

The cells of *Coscinodiscus wailesii* were treated using nickel ions (Ni^2+^) to investigate its effect on the optical characteristics of their frustules [85]. Selected diatom species were grown in sterile filtered seawater containing nickel sulphate (NiSO_4_) at a concentration of 5.0, 1.0, 0.5, and 0.1 mg/L, respectively. The greatest concentration of Ni that had no influence on diatom growth was found to be 0.5 mg/L. The pores in the frustules of Ni-enriched diatoms were more irregular, bigger, and less homogeneous in shape, according to SEM examination. The cytoplasmic morphology of *C. wailesii* grown in the presence of NiSO_4_ revealed thylakoid stack disruption and mitochondrial enlargement. The photoluminescence of silica frustules was quenched as the Ni concentration in the culture media was increased. The EDX method confirmed that the Ni concentration in the diatom frustules was around 0.1% wt. Due to its unique optical properties, diatomaceous biosilica treated with Ni^2+^ can be employed in biotechnology applications.

Zhang et al., 2013 [86], doped biosilica with europium hexavalent nitrate (Eu (NO_3_)_3_.6H_2_O) by cultivating *Navicula* sp. in molar ratio of 1:4 (Eu:Si). The diatom cells were cultured for 96 h, then extracted with ethanol to remove the alcohol-soluble organic material, and the solid remains were heat annealed in air at 1000 °C. The presence of Eu in the forms of Eu_2_O_3_ and Eu_2_SiO_5_ was revealed by XRD analysis. Photoluminescent capabilities were observed in Eu-doped biosilica, with red light emission at 614 nm and excitation at 394 nm, which corresponded to the wavelength of LED emission. These materials can be utilized in fluorescent lamps, plasma display panels, field emission displays, and cathode-ray tubes, among other display technologies.

Similarly, Basharina et al., 2012 [67], studied the effects of zirconium (Zr) and tin (Sn) on the growth, shape, and chemical content of freshwater diatom, *Synedra acus*. To understand their effect on diatom cells, sodium stannate (Na_2_SnO_3_)/zirconium tetrachloride (ZrCl_4_) have been added directly to the culture medium together with sodium silicate (Na_2_SiO_3_). The molybdate blue technique [111] was used to determine the silicon concentration, and inductively coupled plasma mass spectrometry (ICP-MS) was used to determine the contents of Zr and Sn in the culture medium. It was discovered that doping with Zr and Sn resulted in the frustule’s irregularity with a modest decrease in growth rate and the frustule’s mechanical strength. In another experiment, Gannavarapu et al., 2019 [87], studied the cultivation of *Phaeodactylum tricornutum* employing artificial sea water at pH = 9 with the inclusion of 0.8 mM zirconyl oxychloride-octahydrate (ZrOCl_2_.8H_2_O) in order to create nanoporous diatom-ZrO_2_ composites. An electrochemical sensor for detecting methyl parathion, an organophosphorus insecticide, was successfully developed from the obtained composite. Weatherspoon et al., 2007 [88], demonstrated a compact, continuous, and conformal nanocrystalline SnO_2_ coating on the surface of *Aulacoseira* sp. frustules. APTES condensation and Michaelis addition of glucosamine on the diatom surface led to the growth of a number of surface hydroxyl moieties as anchoring groups of SnO_2_. By using a sol–gel technique based on their exposure to tin (IV) 2-propoxide, 2-propanol, and ammonium hydroxide solutions, the OH-enriched frustules were coated with a SnO_2_ layer more efficiently than their bare equivalents. The resultant material was used to build SnO_2_ sensors for NO gas after being annealed at 700 °C.

## 3. Capability for Heavy Metal Uptake and Removal

In terms of heavy metal toxicity, diatoms are one of the most studied species compared to green and blue green algae [46]. To combat heavy metal toxicity, diatoms have been utilized to develop diverse methods such as biotransformation, biomineralization, bioaccumulation, and biosorption [112,113]. Diatoms are good at acquiring and storing metal because the frustule, or silica cell wall, is a tough layer composed of amorphous silica adorned with nano- to micro-sized pores, as well as spines, hyaline region, metal binding surface functional groups and other features that provide a broad contact surface, making heavy metal adsorption easier [46].

To improve the adsorption effectiveness in aqueous media, organosilanes or metal oxides that may covalently link or electrostatically attract positively charged metal species were commonly utilized to functionalize the DE surface. In recent years, the adsorption of heavy metals (such as mercury, chromium, arsenic, silver, lead, copper, cadmium, zinc) by raw/modified DE has been extensively studied [114,115,116,117,118,119,120,121,122]. The porous walls of diatoms after calcination generate many potential sites for adsorption of heavy metals, which in turn allow the simultaneous removal of the cations in solution. These cations can then be easily discharged with weak acid solutions, which enhance their reuse. Various metals removal by diatom frustules have been summarized in Table 2. 

### 3.1. Arsenic

Since arsenate is comparable to phosphate in size, geometry, and ability to engage metabolic activities, diatoms take up dissolved arsenic (As) from the solution. As intake is connected to the prevalent phosphate concentration, according to the previous studies on As accumulation by diatoms and macroalgae. Low quantities of phosphorus have little effect on As uptake. As phosphate uptake rises, As uptake rises as well, until a threshold value is reached, at which point As uptake is prevented [137]. Enhanced phosphate metabolism and selective arsenate uptake are responsible for increased As uptake as phosphate uptake rises at low phosphate concentration. In the case of As adsorption, cation exchange may play a significant role [126]. Weak acid solutions can easily discharge these cations, allowing them to be reused. In another experiment, Thakker et al., 2015 [124], created diatom–iron oxide hybrid as a new sorbent for the removal of As from water by performing overnight reaction between *Phaeodactylum* *tricornutum* culture and ferric chloride solution (FeCl_3_.8H_2_O). The diatom’s porous design was used to immobilize iron oxide and to create a diatom–FeOx composite with a large surface area like 70 m^2^/g. The Langmuir, Freundlich, and D-R models were used to calculate the adsorption mechanism for As^3+^ and As^5+^. According to the Langmuir monolayer adsorption model, the maximum ability of As^3+^ and As^5+^ adsorption by the composite was 10,000 and 12,500 µg/g, respectively. The Freundlich and D-R model described the adsorption process as chemisorptions. Chemically modified biosilica (2 g/L) of *Melosira* sp. with thiol and amino groups showed a 10.99 mg/g As^3+^ adsorption capacity within 26 h of reaction with 2 mg/L As^3+^ at pH 4 [123]. This research has paved the way for further research on development of bio-derived smart materials for environmental cleanup.

### 3.2. Chromium

Hedayatkhah et al., 2018 [125], used two diatoms viz. *Phaeodactylum tricornutum* and *Navicula pelliculosa* for their potential to bioremediate dichromate while simultaneously creating lipids that can be used as biofuel. The strains’ dichromate resistance was tested under various growing conditions in order to achieve large biomass yields, lipid accumulation, and dichromate elimination from the medium. In medium supplemented with 1 mg/L of dichromate, both algal strains thrived well, and biomass yield was enhanced. Dichromate expulsion from the medium was found to be favorably linked with biomass yield under various growing conditions. The elimination of dichromate by living cells was comparable to the autoclaved dead cells or isolated extracellular polymeric compounds (EPS). It was also predicted that dichromate biosorption to cell-associated polymeric compounds was the primary bioremediation mechanism.

In a separate experiment, Hernández-Avila et al., 2017 [126], demonstrated that diatoms are highly efficient in cationic metal exchange, with the exception for hexavalent chromium (Cr^6+^), where exchange rate was just 9%. This could be because Cr^6+^ can be decreased from Cr^6+^ to Cr^3+^ in a reducing environment, promoting its exclusion from solutions. Cr^6+^, on the other hand, exceeds its ability of reduction at high concentrations, preventing adequate removal [138]. As a result, the concentrations of all cations are comparable, and there is no reducing environment. Cr^6+^ could not be converted to Cr^3+^, owing to limited exchange efficiency of diatom for this cation.

### 3.3. Mercury

Yu et al., 2012 [127], chemically modified diatom-based biosilica microparticles with self-assembled monolayers of 3-mercaptopropyl-trimethoxysilane (MPTMS), APTES, and n-(2-aminoethyl)-3-aminopropyl-trimethoxysilane (AEAPTMS) for the adsorption of mercury ions (Hg*^2+^),* respectively. The functional groups (–SH or–NH_2_) were successfully transplanted onto the surface of diatom silica as confirmed from FTIR and x-ray photoelectron spectroscopy investigations. Chemical modifications of silica microparticles significantly increased the kinetics and efficiency of Hg*^2+^* adsorption. Biosilica functionalized with MPTMS/APTES/AEAPTMS achieved equilibrium for Hg*^2+^* adsorption at 60 min of reaction, with maximal adsorption capacities of 185.2, 131.7, and 169.5 mgg^−1^ for MPTMS, APTES, and AEAPTMS, respectively. A pseudo-second-order reaction model and a Langmuirian isotherm were used to explain the adsorption behavior. These findings suggested that diatom-based silica microparticles with mercapto- or amino-functionalized surfaces were promising, natural, cost-effective, and environmentally friendly adsorbents for removing Hg*^2+^* from aqueous solutions.

Later on, Kabiri et al., 2015 [128], prepared a self-assembled aerogel using graphene sheets and FeOOH NPs decorated DE. It was found to be an effective adsorbent for Hg*^2+^* removal from high concentration levels, with an adsorption capacity of >500 mg/g (at 400 mg/L Hg^2+^) of Hg^2+^, outperforming many competing adsorbents. Based on its outstanding adsorption performance, this new adsorbent synthesized by a simple procedure using two low-cost natural minerals widely available from the mining sector (graphite and DE) is a viable solution for the creation of efficient and cost-competitive adsorbents. The Langmuir model fit nicely with the Hg*^2+^* adsorption isotherm. The produced aerogels showed excellent adsorption ability for Hg*^2+^* removal from water, which is important for environmental applications.

### 3.4. Silver, Cadmium, Lead, Copper

Diatomite is an excellent natural resource to remove silver ions (Ag^+^) by cationic exchange from aqueous solution, reported by Hernández-Ávila et al., 2017 [126]. The biosilica of two different diatoms with same physico-chemical behavior showed efficacy in elimination of 95% Ag^+^ ions from a synthetic solution containing 4.280 mg Ag^+^/L. The 95% of silver recovery was confirmed through atomic absorption spectroscopy (AAS) by biosilica after 7 days of exposure in a silver nitrate solution. The authors also mentioned that calcination of diatomite improved their ionic exchange capacity and the heat treatment increased the cations absorption ability. It was observed by Hernández-Ávila et al., 2017 [126], that heavy metal filtering could be possible by biosilica due to their high retention capacity as long as the metal concentration are not too high, and the solution is not too acidic.

It was discovered that frustule layer protein-frustulins, showed a response to environmental stress [134]. The extracellular polysaccharides were reduced and the amount of frustulin proteins was enhanced (by 6 times) after exposing *Nitzschia palea* to Cd. Since the majority of Cd were attached to frustulins (85.4%), it was observed that this metal was mostly retained extracellularly. In another study Khraisheh et al., 2004 [129], removed heavy metal from wastewater using a manganese oxide modified-diatomite (Mn-diatomite) complex. The diatomite impregnated with 0.38 g g^−1^ manganese oxide showed a 2.4-fold increased surface area for removing lead (Pb^2+^), copper (Cu^2+^), and Cd^2+^ in comparison to untreated diatomite. The diatoms *Nitzschia palea* and *Navicula incerta*, immobilized in calcium–alginate beads, showed high Pb^2+^ and Cd^2+^ removal capacities from aqueous solutions [130]. A large amount of Cu^2+^ adsorption by freshwater diatom, *Navicula subminuscula*, and seawater diatoms, *Thalassiosira weissflogii* and *Phaeodactylum tricornutum*, has been documented by Cherifi et al., 2016 [131] and Gonzalez-Davila et al., 2000 [132], respectively. Zhou et al., 2022 [139], reported that sufficient availability of nitrogen and phosphorus in the surroundings induced maximum Cu adsorption by *Phaeodactylum tricornutum*. In another report, maximum Cu uptake by *Planothidium lanceolatum* has been observed at pH 6.0 with an increasing temperature from 15 to 25 °C [133].

### 3.5. Zinc and Iron

Ellwood and Hunter, 2000 [135], cultured *Thalassiosira pseudonana* in a sea water medium supplemented with Zn or Fe salts to verify whether or not diatoms could incorporate significant amounts of Zn and Fe within its cells. There was a sigmoidal relationship between the content of free Zn^2+^ and uptake of Zn^2+^ by biosilica. On the other hand, Fe uptake did not increase with the increase of Fe concentration in culture media. Jaccard et al., 2009 [136], corroborated the research findings of Ellwood and Hunter, 2000 [130]. They grew *Stephanodiscus hantzschii* in a modified medium [140] with a Zn–EDTA complex. ICP-MS confirmed the presence of Zn^2+^ in the structure of diatom frustules. The amount of Zn^2+^ integrated into the diatom frustules was not specified; however, it was discovered that at a concentration of 10^−8.5^ M Zn^2+^, the maximum degree of Zn^2+^ incorporation was reached. Biosorption of 104 and 127 mg of Zn^2+^ from aqueous solution of zinc chloride (pH-8) was confirmed while using 16 × 10^8^ diatom cells/ L of *Planothidium lanceolatum* [133] and *Navicula subminuscula* [131] as biosorbents, respectively. The pennate diatom, *Fragilariopsis* sp. from West Antarctica, has been identified as an efficient Fe removal agent because of its ability for Fe incorporation into biosilica [141].

## 4. Conclusions

The idea of using diatoms in nanobiotechnology has been around for nearly 30 years [142,143], and since then, biosynthesis of nanoparticles using these microorganisms has gained constantly growing attention. In this review, we have summarized the potential of diatom-derived biosilica as a solid support of metal nanomaterials and presented the current trends in this field. With such an intricate natural variability of frustule design, renewability and reproducibility of 3D structures, diatoms emerge as a serious candidate in a low cost and eco-friendly production of various metal particles with innumerable applications.

Nowadays, a metal-doped diatom biosilica can be successfully used (see Table 1 and Table 2) in anticancer treatment, due to biocompatibility, nanoscale pore structure and filtration property; fabrication of wide range biosensing devices, due to multifarious optical properties; bioremediation and water purification, due to heavy metals resistant ability, availability in the local ecosystems, high adsorption (biosorption) and absorption capabilities of diatom frustules. The list of diatom-based nanocomposites and their potential usages seems endless.

Siliceous solid support is always beneficial for any metal particles to increase their stability, and diatom is abundantly available in aquatic environments as silica producing nano-factories to provide low-cost silica. There are certain challenges to utilize diatom-based biosilica in nanobiotechnology like removal of other contaminants from naturally occurring diatoms, establishment of monoculture in laboratory condition, establishment of large-scale diatom cultivation in laboratory condition, difficulties in the cleaning of diatom frustules to obtain only siliceous nanostructures without any trace of other cellular compounds, etc. However, in our opinion, it is possible to construct frustule-doped metal particles within a short period of research work, as diatom shows a high growth rate and high metal uptake capacity. Therefore, it can be concluded that photonic diatom-based biosilica would be the best option to construct any metal–silica nanohybrid following an eco-friendly route in the future.

## Figures and Tables

**Table 1:** Showing summary of the applications of metal associated diatom-based biosilica.

Metals	Source of Silica	Mode of Synthesis	Applications of Metal Conjugated Silica	References
Ag	*Halamphora subturgida*	diffusion-limited aggregate (DLA) model	biosensor	[60]
	*Pinnularia* sp.	AgNPs coating on frustules using APTES adhesive	SERS sensing	[61]
Au	diatomite	PEG altered diatomite being adorned with AuNPs by one-pot liquid-phase synthesis	considered as safe material for medical applications as showed less cytotoxic effect on HeLa cells after 72 h of incubation	[62]
	*Stephanopyxis turris* *Eucampia zodiacus* *Thalassiosira pseudonana*	covalent coupling method	being exploited as favorable catalyst for oxidation of d-Glucose to d-gluconic acid.	[63]
	diatom derived biosilica	electroless Au deposition onto a diatom silica substrate, following substrate expulsion by acid dissolution, allows for the creation of self-supporting gold microstructures.	used as catalyst in reduction of 4-nitrophenol to 4-aminophenol in the presence sodium borohydride	[64]
	*Aulacoseria* sp.	chemically modified frustules being decorated with AuNPs by Ex situ and In situ method	gentamicin delivery in simulated body fluid	[65]
Ti	*Pinnularia* sp.	utilizing a two-stage photobioreactor cultivation procedure, Ti was metabolically inserted into the diatom’s patterned biosilica	biocompatible dye-sensitized solar cells (DSSCs)	[59]
	*Thalassiosira weissflogii*	chemical modification of frustule in vivo by TiBALDH	metabolic substitution with silicon for a dopant variety template with better photocatalytic activity	[66]
	*Coscinodiscus wailesii* and *Synedra acus*	in vivo doping of Ti through addition in the culture media	metabolic substitution with silicon for a dopant variety template	[67,68]
	*Fistulifera solaris*	chemical modification of frustules in vivo by TiBALDH	metabolic substitution with silicon for a dopant variety template, would be useful in bioremediation, water purification, and energy conversion/storage.	[69]
Ge	*Pinnularia* sp.	metabolic insertion of Ge into the frustules through culture medium	imparts optoelectronic properties	[70,71]
	*Nitzschia frustulum*	metabolic insertion of Ge into the frustules through culture medium	nanocomb structures with optoelectronic properties	[72]
	*Thalassiosira pseudonana*	metabolic insertion of Ge into the frustules through culture medium	Fabrication of electronic devices	[73]
Pd	Diatomite	chemically modified diatomite in presence of PVP	used as catalyst in Heck and Suzuki reactions	[74]
	*Pseudostaurosira trainorii*	ultrasound treatment of frustules with PdCl_2_	showed methyl orange removal efficiency	[75]
Pt	*Coscinodiscus wailesii*	diatom-templated Pt by layer-by-layer deposition and covalent linking	high catalytic activity in redox reaction between hexacyanoferrate (III) and thiosulfate	[76]
Fe	Diatomite	dopamine-modified Fe_3_O_4_ nanoparticle self-assembled on the diatom surface in one step by electrostatic attraction	supercapacitors, drug delivery	[27,77]
Mn	Diatomite	etching process.	supercapacitors	[77]
Cd	*Pinnularia* sp.	using chemical bath deposition process, nanostructured polycrystalline CdS thin film coated on biosilica substrate	chemical sensor	[78]
Zn	*Coscinodiscus lineatus*	deposition of ZnS onto frustules by sonochemical process	photonic applications	[79]
	Diatomaceous earth	coating of Zn particles on diatom frustules by exposing to an acetate precursor solution	photonic applications	[80]
Al	*Stephanopyxis turris*	in vivo doping of Al through addition in the culture media	-	[81]
	*Thalassiosira pseudonana*	in vivo and in vitro doping of Al through addition in the culture media	strong catalytic activity	[82]
Ca	*Thalassiosira weissflogii* and *Coscinodiscus* sp.	in vivo doping of Cd through addition in the culture media	would be a useful substrate for the development of fibroblasts and osteoblasts	[83,84]
Ni	*Coscinodiscus wailesii*	doping of Ni through addition in the culture media	chemical sensor	[85]
Eu	*Navicula* sp.	doping of Eu through addition in the culture media	would be utilized in fluorescent lamps, plasma display panels, field emission displays, and cathode-ray tubes	[86]
Zr	*Phaeodactylum tricornutum*	doping of Zr through addition in the culture media	electrochemical sensor	[87]
Sn	*Aulacoseira* sp.	an automated surface sol-gel method was used to coat the hydroxy-rich diatom frustules in SnO_2_	chemical sensor	[88]

**Table 2:** Metal remediation by diatom based biosilica.

Metal	Source of Biosilica	Mode of Remediation	Metal Removal Capacity	References
Arsenic	Diatomite	adsorption, cationic exchange	10.99 mg/g from 2 mg/L As^3+^	[123]
	*Phaeodactylum tricornutum*	diatom–FeOx hybrid mediated adsorption	12,500 µg/g	[124]
Chromium	*Phaeodactylum tricornutum*, *Navicula pelliculosa*	cell-associated polymeric compounds mediated biosorption	1 mg/L	[125]
	Diatomite	cationic exchange	≤10% removal	[126]
Mercury	Diatomite	MPTMS/APTES/AEAPTMS modified biosilica mediated adsorption	185.2, 131.7, and 169.5 mg/g for MPTMS, APTES, and AEAPTMS functionalization, respectively	[127]
	Diatomaceous earth	self-assembled aerogel of graphene sheets and FeOOH NPs decorated DE assisted adsorption	>500 mg/g (at 400 mg/L Hg^2+^) of Hg*^2+^*	[128]
Silver	Diatomite	cationic exchange	95% Ag^+^ from 4.280 mg Ag^+^/L	[126]
Lead	Diatomite	manganese oxide modified-diatomite (Mn-diatomite complex) mediated adsorption	99 mg/g	[129]
	*Nitzschia palea, Navicula incerta*	diatom immobilized calcium-alginate beads-based metal removal	100, 97, 96% from 0.5, 1 and 2 ppm Pb, respectively	[130]
Copper	Diatomite	manganese oxide modified-diatomite (Mn-diatomite complex) mediated adsorption	56.7 mg/g	[129]
	*Navicula subminuscula*	metal Incorporation through absorption process	90% removal	[131]
	*Thalassiosira weissflogii, Phaeodactylum tricornutum*	metal removal through absorption process	-	[132]
	*Planothidium lanceolatum*	metal removal through absorption process	81 mg/g by 16 × 10^8^ diatom cells L^−1^	[133]
Cadmium	*Nitzschia palea*	frustulin protein mediated adsorption	85.4% removal	[134]
	Diatomite	manganese oxide modified-diatomite (Mn-diatomite complex) mediated adsorption	27 mg/g	[129]
	*Nitzschia palea, Navicula incerta*	diatom immobilized calcium-alginate beads-based metal removal	91, 94.6, and 94.5% for Cd from 0.5, 1 and 2 ppm Cd, respectively	[130]
Zinc	*Thalassiosira pseudonana*	metal incorporation through absorption process	1–3% of the total amount of Zn was taken up by the diatom	[135]
	*Stephanodiscus hantzschii*	metal incorporation through absorption process	-	[136]
